# Description of a Virulent Systemic Feline Calicivirus Infection in a Kitten with Footpads Oedema and Fatal Pneumonia

**DOI:** 10.3390/pathogens14111183

**Published:** 2025-11-19

**Authors:** Martina Magliocca, Luciana Mandrioli, Mara Battilani, Barbara Bacci, Giulia Ballotta, Maral Anjomanibenisi, Lorenza Urbani, Liliana Martella, Veronica Facile, Raffaele Scarpellini, Irene Ascenzi, Laura Gallina, Andrea Balboni

**Affiliations:** Department of Veterinary Medical Sciences, Alma Mater Studiorum—University of Bologna, Ozzano Emilia, 40064 Bologna, Italy; martina.magliocca2@unibo.it (M.M.); luciana.mandrioli@unibo.it (L.M.); mara.battilani@unibo.it (M.B.); barbara.bacci@unibo.it (B.B.); giulia.ballotta2@unibo.it (G.B.); maral.anjomanibenisi@studio.unibo.it (M.A.); lorenza.urbani2@unibo.it (L.U.); liliana.martella2@unibo.it (L.M.); veronica.facile2@unibo.it (V.F.); raffaele.scarpellini@unibo.it (R.S.); irene.ascenzi2@unibo.it (I.A.); laura.gallina@unibo.it (L.G.)

**Keywords:** cat, immunohistochemistry, Italy, lung, Maine Coon, phylogeny, viral diversity, virome

## Abstract

Feline calicivirus (FCV) is widespread in multi-cat environments and typically causes acute upper respiratory tract disease (URTD). FCV also causes outbreaks of virulent systemic disease (VSD), mainly in adults, with multiple organ involvement. In this study, an FCV-VSD infection was described in a less-one-month-old Maine Coon kitten originating from a cattery where an outbreak of FCV-URTD had previously been reported. After spontaneous death, post-mortem examination as well as histopathological, immunohistochemical, bacteriological and virological investigations were carried out. Pathological findings were consistent with severe pneumonia and cutaneous oedema of the footpads. No concomitant bacterial infection was detected. FCV RNA was detected in several organs and the highest amount of viral RNA was observed in the lung sample, in which the presence of the FCV antigen was confirmed by immunohistochemistry. With the same immunohistochemical technique, the IBA-1 antibody detected sparse alveolar macrophages, the main viral target cell and pulmonary replication site. The nucleotide sequences of the viral ORF2 gene amplified from all positive tissues were identical with each other and phylogeny confirms that highly virulent FCV strains are not distinguishable from FCV-URTD phenotypes. Our findings reinforce the hypothesis that VSD outbreaks can occur even in small populations, due to the high genetic variability of FCV.

## 1. Introduction

Feline calicivirus (FCV) is a non-enveloped RNA virus, classified in the genus *Vesivirus*, family *Caliciviridae*, which is host specific for the *Felidae* family [1]. It is widely diffuse in multi-cat environments, such as shelters and catteries, and the prevalence is proportional to the number of cats living in these environments [2,3,4]. In fact, due to its resistance to common disinfectants and its ability to survive up to 30 days on dry surfaces [5], FCV can be transmitted through indirect as well as direct contact [6].

After the infection, FCV replicates primarily in the oropharynx and then, through a transient viraemia the virus, can spread to many other tissues [4]. In most cases, FCV is shed approximately 30 days after infection. However, few cats, referred to as asymptomatic carriers, may continue to shed the virus beyond this period—potentially for life [1,7]. This continuous viral shedding represents a great epidemiological significance to susceptible animals, since some of these carriers shed high quantities of viral RNA [8,9]. Clinical signs can be various since different strains of FCV were reported to differ in tropism and virulence. In cats, FCV is a common cause of acute upper respiratory tract disease (URTD) characterised by fever, oculonasal discharge, sneezing and oral ulcerations [1]. However, FCV infection was reported even in association with feline chronic gingivostomatitis [10], limping syndrome [11] and signs of enteritis [12]. Some FCV strains are highly virulent and may cause clinically distinct outbreaks of virulent systemic disease (FCV-VSD), especially in adults, with a high mortality rate. In FCV-VSD, multi-organ involvement is reported, with vasculitis, cutaneous oedema, multifocal ulceration of the skin and footpads, jaundice and pneumonia [13,14,15]. Due to FCVs genomic plasticity, vaccination does not prevent the diffusion of the virus or the emergence of highly virulent strains [16,17]. Therefore, both vaccinated and unvaccinated animals can become infected carriers and can show severe clinical signs [18,19]. VSD outbreaks usually occur after the introduction in the environment of new animals [16], but they can occur also as a result of genetic drift or recombination events related to the variability of the viral RNA genome [3,13]. For this reason, VSD diagnosis is difficult and is mainly pursued by evaluating the clinical presentation, spread and persistence of the virus in the environment and the detection of the same virus in different tissues [16,20].

In this study, the first FCV-VSD infection in a less-one-month-old kitten with footpad oedema and pneumonia was described; genetic characterisation of FCV identified in different biological matrices and tissues was carried out to explore the virome and evaluate viral diversity.

## 2. Materials and Methods

On 4 January 2024, a 20-days-old female Maine Coon kitten (Lab ID: 24/2024) was presented to the Veterinary Teaching Hospital (VTH) of the University of Bologna (Italy), reporting severe dyspnoea and swollen footpads. The kitten was part of a litter of four kittens (two females and two males) and came from an Italian Maine Coon cattery in the province of Bologna (Emilia Romagna region, Italy), in which a previous suspected outbreak of FCV-URTD infection was recorded. The mother was regularly vaccinated. At the time of the kitten’s presentation, the mother and the sister did not show clinical signs. Additionally, the two male kittens showed both swollen footpads and cutaneous lesions in limbs, and one showed oral ulcers. After five days, the two brothers recovered, while the sister developed severe and fatal pneumonia.

After the spontaneous death of the kitten (the same day of the presentation, 4 January 2024), a complete post-mortem examination was carried out within 24 h. Sections of the lung, upper respiratory tract (including trachea and larynx), spleen, liver, heart, kidney, brain, bone marrow, right forelimb, left forelimb, right hind limb and left hind limb, along with conjunctival and oropharyngeal swab samples, were collected. For pathological analyses, tissue samples were fixed in 10% buffered formalin, embedded in paraffin, sectioned at 4 μm and stained with haematoxylin and eosin (HE). Giemsa staining (Histo-Line laboratories, Milan, Italy) to detect mast cells was performed on the footpad’s sections.

All the tissues underwent immunohistochemistry by using mouse monoclonal anti-Calicivirus antibody [FCV1-43] (American Research Products, ARP, Inc.^TM^, supplied by Antibodies.com, Waltham, MA, USA); sections of the lungs were also stained with goat polyclonal AIF-1/Iba1 antibody NB100-1028 (Novus Biologicals, Bio-Techne SRL, Milan, Italy) to detect alveolar macrophages, the viral target cells undergoing diffuse alveolar damage [21]. Briefly, the main passages for both antibodies were the following: endogenous peroxidase inhibition with 3% H_2_O_2_ in methanol; unmasking with citrate solution pH6 with microwave oven at 750 W; preincubation with Bovine Serum PBS 3% BSA 0.25 Tween 20 and with rabbit serum in 20% PBS, respectively; overnight incubation with primary antibody diluted 1:500 with BSA (FCV) and 1:2000 in goat blocking solution (Iba-1); revelation system consisted of biotinylated secondary anti-mouse antibody, chromogen DAB solution kit and Harris and Papanicolaou Haematoxylin counterstain (Histo-Line laboratories, Pantigliate, Milan, Italy), respectively. As positive controls, for FCV, a formalin fixed and paraffin embedded positive tissue of FCV 1072/2018 was used [11]; for Iba-1, a section of canine cutaneous histiocytoma was included. Negative controls, consisting of the omission of primary antibody and replacement with a non-reacting antibody with irrelevant specificity, were also run.

For bacteriological analysis, lung, upper respiratory tract and limb samples were stored at +4 °C for 24 h and were subsequently cultured by streaking on selective (MacConkey agar) and not selective (blood agar, Cled agar and Columbia agar) media at 37 °C in anaerobiosis and aerobiosis conditions. After 24–48 h of incubation, colonies from plates that presented adequate growth were evaluated morphologically and the identification at species level of each isolate was assessed using the matrix-assisted laser desorption–ionisation time-of-flight mass spectrometry method (MALDI-TOF MS) using Bruker Daltonics MBT SMART equipment (Bruker Daltonics, Bremen, Germany), following manufacturer’s instructions and considering a species-level identification when the ID score was >2 (green—high accuracy). If four or more different types of colonies were detected, the growth was considered as non-specific polymicrobism and not further evaluated.

For virological investigations, all tissue and swab samples were stored at −80 °C until analysis and viral RNA was extracted using the RNeasy Mini kit (Qiagen, Hilden, Germany) and the QIAamp Viral RNA Mini Kit (Qiagen, Hilden, Germany), respectively. The RNA extracted was stored at −80 °C until investigation. The presence of FCV in viral RNA was detected using a specific SYBR Green real-time qPCR (qPCR) assay with primers qFCVf (5′-TAA TTC GGT GTT TGA TTT GGC CTG GGC T-3′) and a qFCVr (5′-CAT ATG CGG CTC TGA TGG CTT GAA ACT G-3′) amplifying a fragment of 83 nucleotides (nts) of the ORF1 region [22]. The Power SYBR Green RNA-to-CT 1-Step kit (Life Technologies, Carlsbad, CA, USA) was used in a total volume of 20 μL, following the manufacturer’s instructions. Serial 10-fold dilutions of a plasmid (pCR4 plasmid, TOPO TA Cloning Kit, Life Technologies, USA) containing one copy of the target sequence were used as external standards for the construction of a standard curve. The melting experiment for the evaluation of the specificity of each reaction was performed after the last extension step by a continuous increment from 60 °C to 95 °C. Specific melting temperature was about 81 °C and the limit of detection (LOD) of the assay was one copy of target amplicon/µL of RNA extract (copies/µL). For the FCV identified, reverse transcription was carried out using the SuperScript IV VILO MasterMix (Thermo Fisher Scientific, Waltham, MA, USA) and the 3′ fragment of the ORF2, containing the hypervariable E region, was amplified and sequenced, as previously reported by Balboni and colleagues [11]. Briefly, primers FW4 (5′-CCT GAT GGT TGG CCA GAC AC-3′) and FR4 (5′-GTA CCC TTT GCT CAA GAA TTT TGT-3′) [20] and a Phusion Hot Start II High-Fidelity DNA Polymerase kit (Thermo Fisher Scientific, Waltham, MA, USA) were used to produce amplicons of about 950 nts in length. The amplicons obtained were sequenced by the Sanger method using both forward and reverse primers and the generated nucleotide sequences were assembled, translated into amino acid sequences and aligned with 89 reference sequences from the GenBank database (https://www.ncbi.nlm.nih.gov/genbank/, accessed on 27 November 2024) using the ClustalW method implemented in BioEdit software version 7.2.5 (Tom Hall, Ibis Biosciences, Carlsbad, CA, USA). The assembled FCV sequences were analysed with the BLAST web interface (https://blast.ncbi.nlm.nih.gov/Blast.cgi, accessed on 27 November 2024) and phylogeny was carried out using MEGA version 11.0.10 [23]. A Maximum Likelihood phylogenetic tree of the 3′ fragment of ORF2 of the FCV sequenced in this study and the reference sequences obtained in the GenBank database were constructed using the General Time Reversible model with gamma distribution and invariable sites. One thousand replicates of bootstrap analysis were performed to evaluate the robustness of the phylogenetic tree. Given the clinical signs and pathologic lesions, a possible feline leukaemia virus (FeLV) infection was also considered as a causative agent of immunosuppression and a spleen sample was tested for the presence of FeLV DNA using a specific SYBR Green qPCR assay, as reported by Gallina and colleagues [24].

## 3. Results

**Figure 1:**
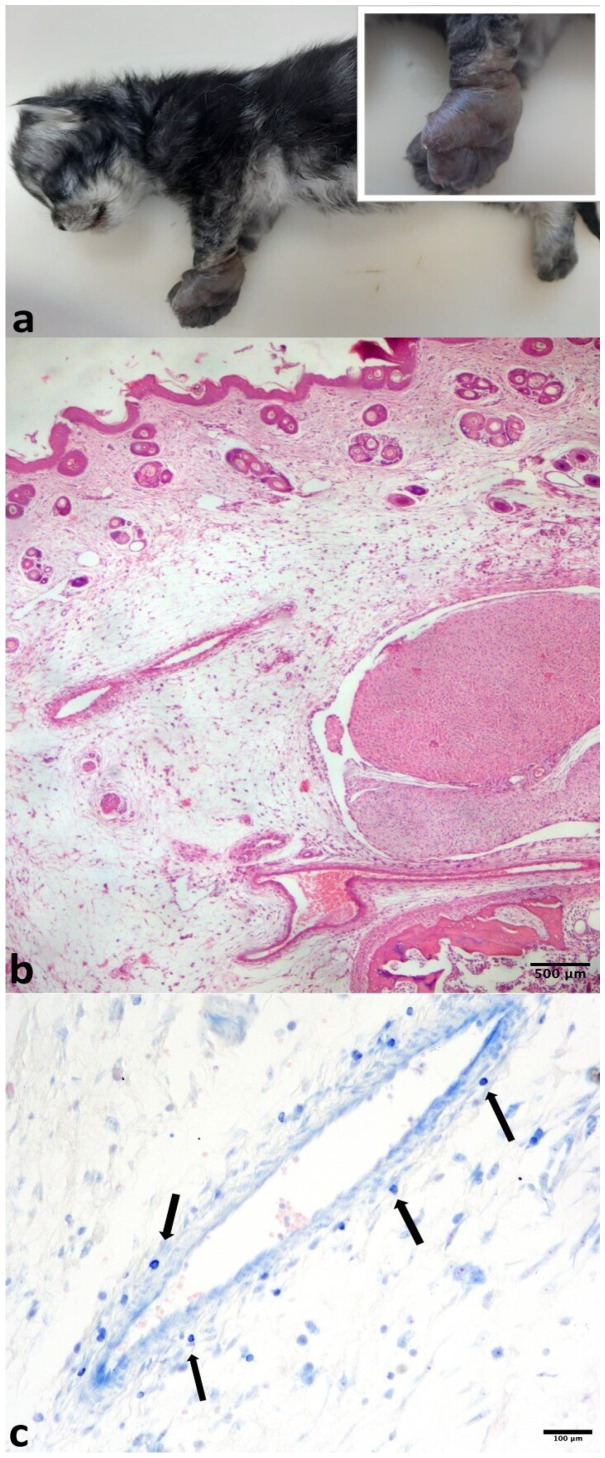
(**a**) Kitten, whole body. Oedematous footpads (better shown in the inset). (**b**) Histology, footpad. Marked, diffuse cutaneous oedema, mainly located in the dermis and the subcutaneous tissue HE. (**c**) Sparse metachromatic mast cells (arrows) passing from the vessel lumen to the vessel wall towards the extracellular matrix, Giemsa.

**Figure 2:**
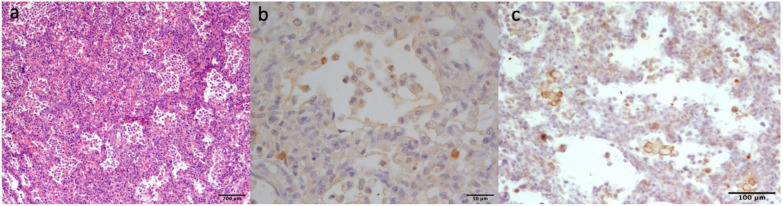
Lung, histology and immunohistochemistry. (**a**) Severe, diffuse interstitial pneumonia (HE). (**b**) Antibody anti-FCV displaying multifocal, cytoplasmic immunoreactivity of rare macrophages. (**c**) Sparse IBA-1 immunoreactive alveolar macrophages.

Post-mortem examination on kitten 24/2024 revealed severe oedema of the footpads (Figure 1a) and intensely and diffusely reddened lungs with an elastic texture. Gross findings in the other organs were unremarkable. Histopathological analysis confirmed diffuse cutaneous oedema in the sections of the footpads (Figure 1b) associated with minimal perivascular dermatitis, which was characterised by the presence of mast cells transmigrating from the vessel walls to the oedematous extracellular matrix (Figure 1c). In the lungs, severe interstitial pneumonia with diffuse alveolar damage and alveolar “desquamation” of macrophages (Figure 2a), multifocal areas of acute haemorrhages and mild fibrin exudation were present. Immunohistochemistry with the anti-FCV antibody labelled the antigen in the cytoplasm of interstitial and alveolar macrophages in the pulmonary tissue (Figure 2b). Sparse macrophages displayed immunoreactivity to the IBA-1 antibody (Figure 2c).

From bacterial isolation, non-specific polymicrobial growth was detected. All samples tested positive to FCV RNA, except bone marrow and conjunctival swab samples, with an overall median quantity of 4 × 10^2^ copies/μL (range 6 × 10^0^–9 × 10^5^) (Table 1). The spleen sample tested negative for FeLV DNA.

**Figure 3:**
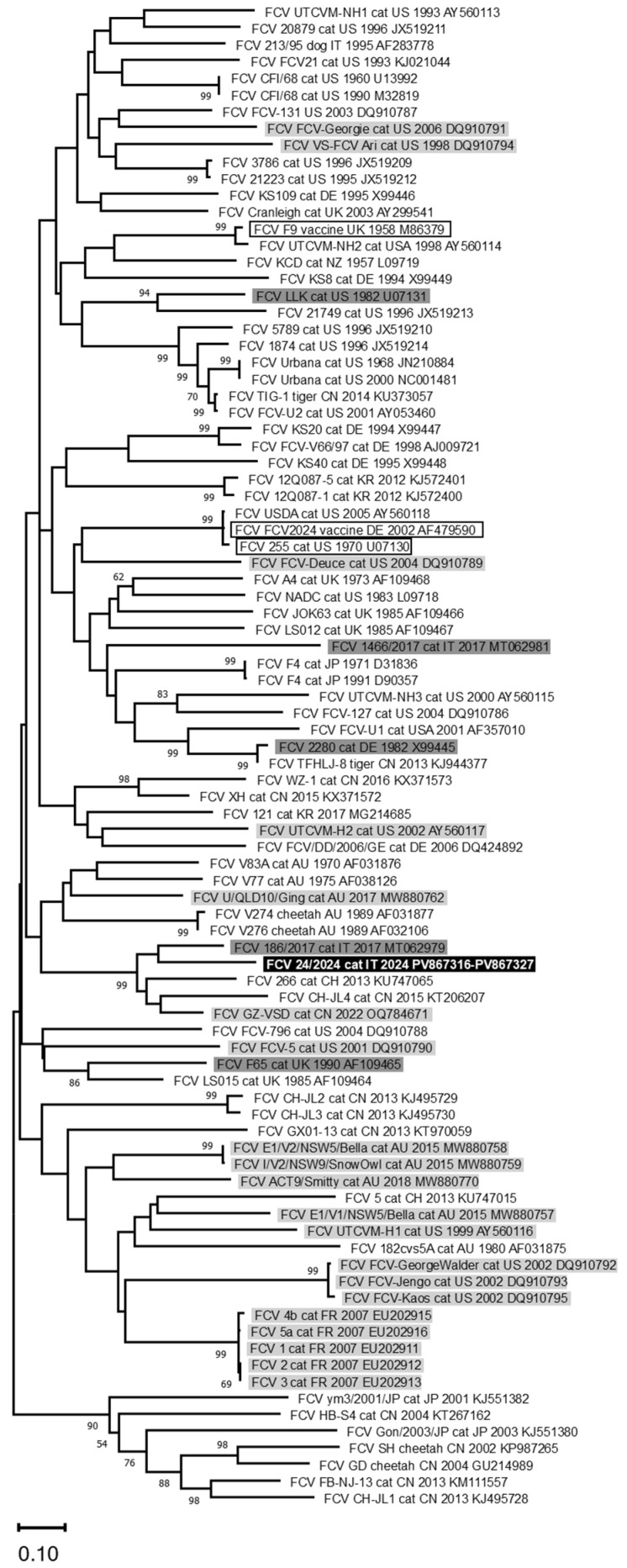
Phylogenetic tree constructed with FCV nucleotide sequences generated in this study and reference sequences available from GenBank of the 3′ fragment of ORF2. Phylogenetic trees were constructed using the Maximum Likelihood method and the General Time Reversible (GTR) model. Bootstrap values calculated on 1000 replicates and ≥60% are indicated on the respective branches. In bold and highlighted in black: one nucleotide sequence representative of all the FCV sequences generated in this study (GenBank ID: PV867316-PV867327). Framed: vaccine reference strains. Highlighted in light grey: reference strains identified in cats showing virulent systemic syndrome. Highlighted in dark grey: reference strains identified in cats showing lameness.

A fragment of 844 nts of the FCV genome, containing the last 717 nts of ORF2 was sequenced from all the samples that tested positive. The obtained nucleotide sequences were identical to each other and showed an identity ≤ 84.5% with FCV sequences available in the GenBank database and ranged from 82.8% to 70.8% with all the reference sequences analysed in this study. The phylogenetic tree did not show clusterisation based on a clinical, geographical and temporal basis (Figure 3), but the sequences obtained in this study were closer to a FCV strain identified in a cat with lameness syndrome (GenBank ID: MT062979) from Italy.

## 4. Discussion

FCV is a highly contagious virus for cats, which represents one of the main health problems in shelters and catteries [1,2]. In most cases, cats show mild clinical signs, mostly related to the upper respiratory tract [25], but due to mutations or recombination events between strains, more serious diseases may develop [20,26,27]. FCV-VSD infection is usually reported in adult cats living in multi-cat environments [13,28] and occasionally in household cats [16,27]. A higher mortality rate was reported in adults (up to 79%) than in young subjects [4,13,16,29]. The pathogenesis of this condition is still obscure and an immune-mediated contribution is probably involved in adult subjects, while kittens may be less predisposed to severe clinical forms due to a more rapid adaptation of the immune response. In particular, some authors, with an anti-FCV monoclonal antibody, documented the presence of the antigen within affected endothelial and epithelial cells, including the skin and lungs, suggesting a mechanism for lesion development that is at least partly immune-mediated [30].

In this study, the first case of a lethal FCV-VSD infection in a less-one-month-old kitten was reported. Clinical signs, including dyspnoea and footpad oedema, were commonly associated with the FCV-VSD infection, as well as rapid disease progression [4,6,31]. In the literature, oedema was reported in association with the FCV-VSD infection, mainly of the head and limbs [32]. In the present case, the oedema was limited to the skin-subcutaneous tissues of the footpads and distal limbs and constituted the unique external gross finding. Giemsa staining on the footpad’s histological sections, as an indication of mast cells migrating from the lumina to the vessel walls towards the extracellular matrix, was added to the routine HE. Mast cells are elements which actively contribute to oedematous fluid generation. This is in agreement with Foley and colleagues [30], who stated that in the skin of FCV-VSD-infected cats, the three most altered cytokines were MIP-1a, IL-10, and TNF-a and that IL-10 stimulates mast cells. In the current case, the histological findings of the lungs were similar to those previously described, and they refer to virus-induced, diffuse alveolar damage occurring in natural caliciviral infections [21]. These authors discovered that FCV infects and replicates in alveolar macrophages and, to a lesser extent, in type II pneumocytes. They were able to state that, even if infrequent, lung involvement is an important feature of FCV. In these rare cases, alveolar macrophages are the main viral target cell and pulmonary replication site, and their infection associated with desquamation and activation and eventually death by apoptosis [21]. In our case, macrophages were labelled with the Iba1 antibody, a marker similar to the CD18 employed on frozen sections by Monnè Rodriguez et al. [21].

Molecular investigation revealed the presence of FCV RNA in all samples tested except for bone marrow and conjunctival swab samples. In particular, the highest amount of viral RNA was observed in the lung sample (9 × 10^5^ copies/µL), which showed the most severe lesions, where it was possible to confirm the presence of the viral antigen by immunohistochemistry. The detection of viral RNA in tissues not typically targeted by FCV supports the hypothesis that the identified virus spread systemically and suggests an infection with a highly virulent viral phenotype. In fact, differently from FCV-URTD viruses, which appear to remain largely confined to the oropharyngeal tract and sporadically extend to the lower respiratory tract [32,33,34], FCV-VSD strains exhibit a great propensity to disseminate in different tissues [35,36].

Viral sequences obtained from all the positive samples were identical to each other, confirming the presence of the same virus in several organs and excluding a possible co-infection between different viral strains. Similar findings were observed by Reynold and colleagues [37], who reported the presence of the same FCV from different tested tissues of two cats affected by VSD. Conversely, Battilani and colleagues [20] reported the presence of viruses genetically and phylogenetically different from each other in the same cat. These opposing findings confirm that FCV has high genetic plasticity, showing significant evolutionary dynamics both within individual hosts and animal populations under favourable circumstances [14,17,38]. Furthermore, the sequences analysed in this study showed an identity of ≤ 84.5% with reference FCV strains, confirming the high genetic variability of this virus [15,38,39]. Conversely, higher nucleotide identity values have typically been found between viruses circulating within the same colony where FCV is endemic [39].

Bacteriological investigation did not reveal any concomitant bacterial infection. Although worsening of clinical conditions is often associated with secondary bacterial infections [40], in this study we can confirm that only FCV was responsible for the rapid disease progression and fatal outcome.

To date, no specific genetic markers that allow for us to distinguish highly virulent strains from common strains have been identified. Consequently, in line with previous studies, in the present case, the phylogenetic tree did not reveal any clustering based on a clinical, geographical and temporal basis [11,41]. In particular, Bordicchia and colleagues [14] identified different FCV-VSD strains from three outbreaks in Australia, which did not cluster phylogenetically with any other FCV-VSD strains previously sequenced. Even Reynolds and colleagues [37] reported no correlation among viruses identified from one outbreak in France with other highly virulent strains. Only viruses which share immediate temporal or spatial links clustered closely [14]. In fact, in the current study, the obtained FCV sequences were genetically closer to a viral strain detected seven years earlier (2017) in a cat with lameness syndrome from the same veterinary hospital (GenBank ID: MT062979) [11], confirming the absence of correlations between clinical forms and phylogeny.

The diagnosis of VSD is challenging, and several authors emphasise that the clinical signs alone are insufficient to draw conclusions. A comprehensive diagnostic approach is required, including assessment of viral dissemination and persistence in the environment, as well as the detection of the same viral strain in multiple tissues [4,16,20,31]. The analyses carried out in the present study support the hypothesis that the kitten, despite its young age, was infected with a highly virulent virus. According to the clinical history, the kitten originated from a Maine Coon cattery where a previous outbreak of FCV-URTD was reported. Although this information was based only on the observation of clinical signs, without confirmation through molecular diagnostic testing, it is possible to suppose that the infected animals may have recovered clinically but subsequently became asymptomatic carriers. In this way, FCV may never have been eliminated from the population, allowing it to gradually accumulate mutations with the emergence of a virulent strain [17]. Pre-existing immunity from vaccination or maternally derived antibodies can reduce or eliminate the onset of clinical signs, but it does not prevent infection [1,7,17]. Furthermore, current vaccine formulations offer limited protection against highly virulent FCV strains, and, as a result, infected animals may still develop severe clinical conditions [16,29,42]. In the present case, although the mother was regularly vaccinated, the possibility that she acted as an asymptomatic carrier and thus contributed to the infection of the entire litter cannot be excluded. The main limitation of this study was the impossibility of conducting more in-depth diagnostic investigations on the rest of the litter and the mother, which would have allowed for an assessment of the epidemiological situation of the cattery. However, the clinical history of the cattery and the clinical signs observed in the two male kittens during the first clinical examination, along with the rapid onset of disease in the sister [29], support the hypothesis that an FCV-VSD strain was circulating in the litter.

The pathogenesis of FCV-VSD infection remains incompletely understood, highlighting the need for ongoing studies to further elucidate its mechanisms and to monitor its epidemiological evolution.

## 5. Conclusions

This study reports the first lethal case of an FCV-VSD infection in a less-one-month-old kitten presenting with severe dyspnoea and swollen footpads. The analyses performed confirm that the severe clinical condition and subsequent death were caused by a highly virulent FCV and support the absence of correlation with other FCV-VSD strains on a clinical, geographical and temporal basis. Clinical history and laboratory findings reinforce the hypothesis that FCV-VSD outbreaks can occur even in small populations, as a result of the high genetic variability of the virus. However, the pathogenesis of the FCV-VSD infection is still unknown and further investigations are needed.

## Figures and Tables

**Table 1:** Samples tested for FCV RNA with respective quantities.

Sample	Target RNA Quantity *	Ct
Lung	9 × 10^5^	18
Right forelimb	3 × 10^2^	31
Left forelimb	5 × 10^3^	26
Right hind limb	2 × 10^2^	31
Left hind limb	2 × 10^2^	31
Spleen	9 × 10^2^	29
Liver	5 × 10^2^	30
Upper respiratory system	3 × 10^2^	30
Heart	1 × 10^3^	28
Kidney	5 × 10^1^	33
Brain	6 × 10^0^	36
Bone marrow	negative	-
Oropharyngeal swab	3 × 10^4^	24
Conjunctival swab	negative	-

Ct: mean threshold cycle. * Mean quantity expressed in copy of target amplicon/µL of RNA extract (copies/µL).

## Data Availability

The original dataset generated and analysed in this study is openly available in AMSActa UNIBO at [https://amsacta.unibo.it/id/eprint/8483/, accessed on 25 August 2025]. The nucleotide sequences generated and analysed during the current study are available in the International Nucleotide Sequence Database Collaboration (INSDC) repository (http://www.insdc.org/; accessed on 2 July 2025 with the IDs: PV867316-PV867327).
